# Significant variation in computed tomography imaging of pregnant trauma patients: a retrospective multicenter study

**DOI:** 10.1007/s10140-023-02195-w

**Published:** 2023-12-27

**Authors:** Alexa N. Lucas, Erika Tay-Lasso, Danielle C. Zezoff, Nicole Fierro, Navpreet K Dhillon, Eric J Ley, Jennifer Smith, Sigrid Burruss, Alden Dahan, Arianne Johnson, William Ganske, Walter L. Biffl, Dunya Bayat, Matthew Castelo, Diane Wintz, Kathryn B Schaffer, Dennis J Zheng, Areti Tillou, Raul Coimbra, Rahul Tuli, Jarrett E. Santorelli, Brent Emigh, Morgan Schellenberg, Kenji Inaba, Thomas K Duncan, Graal Diaz, Katharine A. Kirby, Jeffry Nahmias

**Affiliations:** 1 Department of Emergency Medicine, University of California, San Francisco, San Francisco, CA USA; 2 Division of Trauma, Burns and Surgical Critical Care, Department of Surgery, University of California, Irvine, 3800 W. Chapman Ave., Suite 6200, Orange, CA 92868 USA; 3 Department of Internal Medicine, University of California, Davis, Sacramento, CA USA; 4 Department of Surgery, Cedars-Sinai Medical Center, Los Angeles, CA USA; 5 Division of Trauma and Critical Care, Harbor-UCLA Hospital, Torrance, CA USA; 6 Department of Trauma, Acute Care Surgery, Surgical Critical Care, Loma Linda Medical Center, Loma Linda, CA USA; 7 Riverside School of Medicine, University of California, Riverside, CA USA; 8 Cottage Health Research Institute, Santa Barbara Cottage Hospital, Santa Barbara, CA USA; 9 Trauma and Acute Care Surgery, Scripps Memorial Hospital, La Jolla, CA USA; 10 Department of Surgery, Sharp Memorial Hospital, San Diego, CA USA; 11 Department of Surgery, UCLA David Geffen School of Medicine, Los Angeles, CA USA; 12 Comparative Effectiveness and Clinical Outcomes Research Center (CECORC), Riverside University Health System Medical Center, Riverside, CA USA; 13 Division of Trauma, Surgical Critical Care, Burns and Acute Care Surgery, University of California San Diego School of Medicine, San Diego, CA USA; 14 Division of Acute Care Surgery, LAC+USC Medical Center, University of Southern California, Los Angeles, CA USA; 15 Department of Trauma, Ventura County Medical Center, Ventura, CA USA; 16 Center for Statistical Consulting, Department of Statistics, University of California Irvine, Irvine, CA USA

**Keywords:** Pregnant trauma, Pregnancy, Imaging, Computed tomography, Fetus radiation

## Abstract

**Purpose:**

Following motor vehicle collisions (MVCs), patients often undergo extensive computed tomography (CT) imaging. However, pregnant trauma patients (PTPs) represent a unique population where the risk of fetal radiation may supersede the benefits of liberal CT imaging. This study sought to evaluate imaging practices for PTPs, hypothesizing variability in CT imaging among trauma centers. If demonstrated, this might suggest the need to develop specific guidelines to standardize practice.

**Methods:**

A multicenter retrospective study (2016–2021) was performed at 12 Level-I/II trauma centers. Adult (≥18 years old) PTPs involved in MVCs were included, with no patients excluded. The primary outcome was the frequency of CT. Chi-square tests were used to compare categorical variables, and ANOVA was used to compare the means of normally distributed continuous variables.

**Results:**

A total of 729 PTPs sustained MVCs (73% at high speed of ≥ 25 miles per hour). Most patients were mildly injured but a small variation of injury severity score (range 1.1–4.6, *p* < 0.001) among centers was observed. There was a variation of imaging rates for CT head (range 11.8–62.5%, *p* < 0.001), cervical spine (11.8–75%, *p* < 0.001), chest (4.4–50.2%, *p* < 0.001), and abdomen/pelvis (0–57.3%, *p* < 0.001). In high-speed MVCs, there was variation for CT head (12.5–64.3%, *p* < 0.001), cervical spine (16.7–75%, *p* < 0.001), chest (5.9–83.3%, *p* < 0.001), and abdomen/pelvis (0–60%, *p* < 0.001). There was no difference in mortality (0–2.9%, *p* =0.19).

**Conclusion:**

Significant variability of CT imaging in PTPs after MVCs was demonstrated across 12 trauma centers, supporting the need for standardization of CT imaging for PTPs to reduce unnecessary radiation exposure while ensuring optimal injury identification is achieved.

## Introduction

Trauma affects an estimated 8% of all pregnant patients and is the leading cause of nonobstetric maternal death. Motor vehicle collisions (MVCs) account for half of all injuries in pregnant trauma patients [1, 2]. In the general adult, non-pregnant population, MVCs are considered significant mechanisms of injury, and liberal computed tomography (CT) imaging is often recommended [2–5] to expeditiously identify and properly treat injuries. This is especially important with high-speed MVCs (≥25 miles per hour), which carry substantial risk for immediate life-threatening injuries [1, 2, 4–15].

Adult non-pregnant trauma patients commonly undergo CT imaging of the head, cervical spine, chest, and abdomen/pelvis [1, 2, 4, 5, 7, 8, 12]. In pregnant patients, this carries a risk to the developing fetus. According to the American College of Obstetrics and Gynecology (ACOG), a fetus can receive up to 50 mGy [16] of radiation safely, which would allow for CT imaging of the head, cervical spine, chest, and abdomen/pelvis. Historically, the principle of “benefits to the mother outweighs small risks to the fetus” has guided trauma providers [1, 2, 4, 5, 12, 17, 18] and is professed in the Advanced Trauma Life Support (ATLS) textbook. Therefore, ATLS recommends that the principles of trauma assessment remain the same for the gravid and non-gravid trauma patient [3]. Despite this, some trauma centers have created their own guidelines for the imaging evaluation of pregnant trauma patients (PTPs). However, a report by Shakerian et al. shows compliance to a center’s own imaging guidelines for PTPs can be quite low (only 18% in their single-center report) [19]. In addition, there are no national consensus guidelines defining when to perform CT imaging of PTPs outside of ATLS which professes that PTPs should be treated the same as the non-gravid patient. This suggests PTP imaging is not occurring in a standardized manner across all centers [19], and given there is no data available directly comparing trauma centers, this multicenter study aimed to evaluate CT imaging of PTPs involved in MVCs, hypothesizing significant variability in CT imaging practices among trauma centers. If found, this might indicate the need to better adhere to existing guidelines or develop PTP-specific guidelines to better standardize care and foster best practices.

## Methods

This study was approved by the institutional review board of all participating centers, and the requirement for informed consent was waived. A multicenter retrospective study of adult PTPs sustaining a MVC from 2016 to 2021 at 12 American College of Surgeons-verified Level-I or Level-II trauma centers was performed.

All PTPs 18 years of age or older and evaluated by the trauma team following MVCs were included. No patients were excluded. The primary outcome was CT imaging of the head, cervical spine, chest, and abdomen/pelvis. Secondary outcomes included mortality during index hospitalization, hospital length of stay (LOS), intensive care unit (ICU) LOS, fetal delivery during hospital stay, and in-hospital operations, which included tracheostomy, laparotomy, craniotomy/craniectomy, vascular/endovascular surgery, angioembolization, resuscitative hysterotomy, and perimortem cesarean section.

Demographic information including age and gestational age of the fetus (weeks), as well as Glasgow Coma Scale (GCS) score, injury severity score (ISS), and abbreviated injury scale (AIS) for the head, face, neck, thorax, abdomen, spine, upper/lower extremity, and external regions, was collected.

All categorical variables were coded as yes or no. The data was collected through a centralized *REDCap* data collection tool [20, 21]. The STROBE guideline was used to ensure proper reporting of methods, results, and discussion (SDC 1).

### Data analysis

Summary statistics were used to compare demographics, clinical characteristics, and patient outcomes among trauma centers for PTPs involved in MVCs and separately among a subset of MVCs that were reported at high speed (defined as ≥25 miles per hour). Notably, two trauma centers did not collect information regarding the reported speed of the MVC, so they were excluded from this subset analysis of high-speed MVCs. Means and standard deviations, medians and interquartile ranges, and frequencies and percentages were calculated as appropriate for normally distributed continuous, non-normal continuous, and categorical variables, respectively. The chi-square test was used to compare the distribution of categorical variables across centers. ANOVA was used to compare the means of normally distributed continuous variables across centers, and the Kruskal-Wallis test was performed to test whether the non-normal continuous variables at each center originated from the same distribution. *p*-values of less than 0.05 were considered statistically significant. All analyses were performed by a statistician using Stata software (StataCorp. 2021. *Stata Statistical Software: Release 17*. College Station, Texas—StataCorp LLC).

## Results

### Demographics and injury profile for all PTPs involved in MVCs

A total of 727 PTPs were involved in MVCs, evaluated by trauma teams at ten Level-I trauma centers and two Level-II trauma centers. When comparing centers, PTPs were of a similar age and had a similar gestational age. However, there was a statistically significant difference in median ISS among centers, although the overall population was relatively mildly injured (range 1.1–4.6, *p* < 0.001). Also, the median AIS scores varied across centers for the face (*p* < 0.001), spine (*p* = 0.002), and external/other (*p* < 0.001) regions (Table 1).

**Table 1:** Demographics of pregnant trauma patients involved in motor vehicle collisions at 12 trauma centers

Total, *N*=727	A	B	C	D	E	F	G	H	I	J	K	L	*p*-value^†^
Age (years)	30.9	30.7	27.2	27.3	29.6	27.6	28.2	28.1	26.7	28.1	27.4	28.0	0.1
Gestation age	23 6/7	26 6/7	25 0/7	30 2/7	26 1/7	26 6/7	27 1/7	27 5/7	27 6/7	26 4/7	24 5/7	29 2/7	0.29
GCS, median	15 (11–15)	15 (15–15)	15 (13–15)	15 (15–15)	15 (3–15)	15 (15–15)	15 (14–15)	15 (8–15)	15 (14–15)	15 (10–15)	15 (9–15)	15 (14–15)	0.01^‡^
ISS, score	1 (0–10)	1 (0–5)	1 (0–33)	1 (0–2)	1 (0–34)	1 (0–18)	1 (0–18)	1 (0–29)	1 (0–29)	1 (0–24)	1 (0–22)	1 (0–14)	**<0.001**^‡^
AIS, head	2.5 (2–3)	--	2 (0–3)	1 (1–1)	3 (1–4)	1 (1–2)	0 (0–9)	2.5 (1–4)	1 (1–1)	2 (1–4)	2 (1–3)	2 (0–6)	0.84^‡^
AIS, face	--	--	1 (0–1)	--	1 (1–1)	--	0 (0–1)	1 (1–1)	1 (1–2)	1 (1–2)	1 (1–2)	0 (0–0)	**<0.001**^‡^
AIS, neck	--	--	0 (0–3)	--	--	1 (1–2)	0 (0–1)	--	1 (1–1)	1 (1–4)	1 (1–2)	1 (0–2)	0.06^‡^
AIS, thorax	--	--	2 (0–3)	--	1 (1–3)	1 (1–4)	1 (0–6)	2.5 (2–3)	1 (1–4)	1 (1–1)	2 (1–3)	2 (0–3)	0.85^‡^
AIS, abdomen	2 (2–2)	--	2 (0–5)	1 (1–1)	2 (1–4)	1 (1–1)	0 (0–8)	2 (1–3)	1 (1–3)	1 (1–2)	1 (1–3)	1.5 (1–2)	0123^‡^
AIS, spine	--	--	0.5 (0–3)	--	2 (1–2)	1 (1–1)	0 (0–0)	--	1 (1–1)	2 (1–2)	2 (2–2)	0 (0–0)	**0.002**^‡^
AIS, upper extremity	--	--	0.5 (0–1)	1 (1–1)	2 (1–2)	1 (1–1)	0 (0–5)	2 (2–2)	1 (1–1)	1 (1–2)	1 (1–2)	1 (0–2)	**0.002**^‡^
AIS, lower extremity	2 (2–2)	--	2 (0–4)	1 (1–1)	2 (2–2)	2 (1–2)	0 (0–4)	2 (2–3)	1 (1–2)	1 (1–3)	1 (1–3)	0 (0–0)	0.07^‡^
AIS, external	1 (1–1)	--	1 (0–1)	1 (1–1)	1 (1–1)	1 (1–1)	1 (0–3)	1 (1–1)	1 (1–1)	1 (1–1)	1 (1–2)	1 (1–1)	**<0.001**^‡^

*GCS*, Glasgow Coma Scale; *ISS*, injury severity score; *AIS*, abbreviated injury scale. ^†^*p*-values are from chi-square tests unless noted otherwise. ^‡^Kruskal-Wallis test *p*-value

Median and range unless noted otherwise

### Imaging and outcomes for all PTPs involved in MVCs

There were significant variations in patients undergoing a CT head (11.8–62.5%, *p* < 0.001), CT cervical spine (11.8–75%, *p* < 0.001), CT chest (4.48–50.25%, *p* < 0.001), and CT abdomen/pelvis (11.8–62.5%, *p* < 0.001) among trauma centers. Similarly, there was variation in the use of magnetic resonance imaging (MRI) (0.0–17.6%, *p* = 0.002) (Table 2).

**Table 2:** Imaging practices for pregnant trauma patients involved in motor vehicle collisions at 12 trauma centers

Total, *N*=727	A (12)	B (30)	C (59)	D (8)	E (34)	F (67)	G (199)	H (29)	I (64)	J (133)	K (68)	L (24)	*p*-value^†^
E-FAST positive	0% (0)	3.3% (1)	10.9% (6)	0% (0)	2.9% (1)	6.0% (4)	0.5% (1)	3.4% (1)	1.8% (1)	0.8% (1)	8.8% (6)	0.0% (0)	**0.002**
CXR	8.3% (1)	0% (0)	5.5% (3)	0% (0)	3.0% (1)	0% (0)	1.0% (2)	6.2% (1)	7.8% (4)	1.5% (2)	1.5% (1)	8.3% (2)	0.06
MRI	8.3% (1)	6.7% (2)	3.4% (2)	0% (0)	17.6% (6)	11.9% (8)	4.5% (9)	0% (0)	4.7% (3)	4.5% (6)	17.6% (12)	16.7% (4)	**0.002**
CT head	33.3% (4)	23.3% (7)	33.9% (20)	62.5% (5)	11.8% (4)	11.9% (8)	26.1% (52)	41.4% (12)	28.1% (19)	32.3% (43)	30.9% (21)	62.5% (15)	**<0.001**
CT cervical spine	33.3% (4)	23.3% (7)	37.3% (22)	75.0% (6)	11.8% (4)	20.9% (14)	46.2% (92)	41.4% (12)	32.8% (21)	42.9% (57)	36.8% (25)	58.3% (14)	**<0.001**
CT chest	25.0% (3)	13.3% (4)	28.8% (17)	12.5% (1)	5.8% (2)	4.5% (3)	50.3% (100)	48.2% (14)	21.9% (14)	6.02% (8)	29.4% (20)	29.2% (7)	**<0.001**
CT abdomen/pelvis	16.7% (2)	23.3% (7)	28.8% (17)	0.0% (0)	14.7% (5)	4.5% (3)	57.3% (114)	55.2% (16)	28.1% (18)	8.3% (11)	45.6% (31)	16.7% (4)	**<0.001**

*E-FAST*, extended focused assessment with sonography in trauma; *CXR*, chest radiograph with significant findings; *MRI*, magnetic resonance imaging; *CT*, computed tomography

^†^*p*-values are from chi-square tests unless noted otherwise

There was no difference in overall hospital procedures (0–11.9%, *p* = 0.05) but there was variation in the rate of cesarean hysterectomy (0.0–75.0%, *p* = 0.01). Only three cesarean hysterectomies were performed in total, all of which occurred at one site. There was also a similar rate of fetal delivery among centers. ICU LOS was similar among centers but there was variability in overall LOS (1.1–4.6 days, *p* < 0.001) and ventilator days (0.0–0.3 days, *p* = 0.01), although ventilator days were rare. Finally, there was no difference in mortality (0.0–2.9%, 0.19) when comparing trauma centers (Table 3).

**Table 3:** Outcomes for pregnant trauma patients involved in motor vehicle collisions at 12 trauma centers

Total, *N*=727	A	B	C	D	E	F	G	H	I	J	K	L	*p*-value^†^
Required in-hospital operations	8.3% (1)	0.0%	11.9% (7)	0.0%	2.9% (1)	1.5% (1)	2.0% (4)	10.3% (3)	6.2% (4)	4.5% (6)	7.4% (5)	0.0%	0.05^§^
Tracheostomy	0.0%	0.0%	0.0%	0.0%	0.0%	0.0%	0.0%	0.0%	0.0%	0.0%	0.0%	0.0%	n/a^§^
Laparotomy	0.0%	0.0%	3.4%	0.0%	2.9%	0.0%	0.5%	3.5%	4.7%	0.0%	0.0%	0.0%	0.08^§^
Thoracotomy	0.0%	0.0%	0.0%	0.0%	0.0%	0.0%	0.0%	0.0%	0.0%	0.0%	0.0%	0.0%	n/a^§^
Craniotomy/craniectomy	0.0%	0.0%	0.0%	0.0%	0.0%	0.0%	0.0%	3.5%	0.0%	0.0%	0.0%	0.0%	0.19^§^
Vascular/endovascular surgery	0.0%	0.0%	0.0%	0.0%	0.0%	0.0%	0.0%	3.5%	1.6%	0.8%	0.0%	0.0%	0.51^§^
Resuscitative hysterotomy/perimortem C-section	0.0%	0.0%	1.7%	0.0%	0.0%	0.0%	0.0%	0.0%	0.0%	0.0%	0.0%	0.0%	1
Cesarean hysterectomy	0.0%	0.0%	0.0%	0.0%	0.0%	0.0%	0.0%	0.0%	4.7%	0.0%	0.0%	0.0%	0.01
Fetal delivery during hospital stay	8.3% (1)	3.3% (1)	5.1% (3)	0%	11.8% (4)	6.0% (4)	7.0% (14)	10.3% (3)	7.8% (5)	4.5% (6)	10.3% (7)	8.3% (2)	0.87
ICU days, median (range)	0 (0–3)	0 (0–0)	0 (0–3)	0 (0–3)	0 (0–8)	0 (0–13)	0 (0–2)	0 (0–6)	0 (0–4)	0 (0–6)	0 (0–4)	0.0	0.03^‡^
Ventilator days, median (range)	0 (0–2)	0 (0–0)	0 (0–3)	0 (0–0)	0 (0–4)	0 (0–0)	0 (0–0)	0 (0–8)	0 (0–2)	0 (0–1)	0 (0–0)	0 (0–0)	**0.03**^‡^
Length of stay in days, median (range)	2 (1–9)	1 (0–4)	1 (1–76)	2 (1–2)	1 (1–15)	1 (0–12)	2 (0–65)	2 (1–68)	1 (0–6)	1 (0–10)	2 (1–24)	1 (0–6)	**<0.001**^‡^
Mortality	0.0%	0.0%	0.0%	0.0%	2.9% (1)	0.0%	0.0%	0.0%	0.0%	0.0%	0.0%	0.0%	0.19^§^

*C-section*, cesarean section; *ICU*, intensive care unit

^†^*p*-values are from chi-square tests unless noted otherwise. ^‡^Kruskal-Wallis test *p*-value, ^§^Fisher’s exact test *p*-value

### Demographics and injury profile for PTPs involved in high-speed MVCs

Of the 729 PTPs, 480 (65.8%) were involved in high-speed MVCs. In this cohort, there were no differences in age or gestational age among centers. The median ISS varied among centers, although this high-speed MVC population was relatively mildly injured (range 1.0–4.7, *p* < 0.001). The median AIS scores were similar among centers, except for the face and external regions (Table 4).

**Table 4:** Demographics for pregnant trauma patients involved in high-speed (>25 mph) motor vehicle collisions at ten trauma centers

Total, *N*=480	C*	D	E	F	G	H	I	J	K	L	*p*-value^†^
Age (years)	27.1	27.3	29.8	27.2	28.2	28.6	26.0	28.3	26.5	27.6	0.1
Gestation (weeks + days/7)	25 2/7	30 2/7	25 4/7	27 1/7	26 6/7	25 5/7	28 1/7	26 4/7	23 6/7	31 6/7	0.14^‡^
GCS	15 (13–15)	15 (15–15)	15 (3–15)	15 (15–15)	15 (14–15)	15 (8–15)	15 (14–15)	15 (10–15)	15 (9–15)	15 (14–15)	0.09^‡^
ISS, score	1 (0–33)	1 (0–2)	1 (1–34)	1 (1–18)	1 (0–18)	1 (0–29)	1 (1–29)	1 (1–21)	1 (0–22)	1 (1–5)	**<0.001**^‡^
AIS, head	2 (0–3)	1 (1–1)	3 (1–4)	1 (1–2)	0.5 (0–9)	2.5 (1–4)	1 (1–1)	2 (1–2)	2 (1–3)	2 (0–6)	0.22^‡^
AIS, face	1 (0–1)	--	1 (1–1)	--	0 (0–1)	1 (1–1)	1 (1–1)	1 (1–2)	1 (1–2)	0 (0–0)	**0.01**^‡^
AIS, neck	0 (0–3)	--	--	1 (1–2)	0 (0–1)	--	1 (1–1)	1 (1–4)	1 (1–2)	1 (0–2)	0.26^‡^
AIS, thorax	2 (0–3)	--	1.5 (1–3)	2.5 (1–4)	2 (0–6)	3 (2–3)	1.5 (1–4)	1 (1–1)	2 (1–3)	1 (0–2)	0.70^‡^
AIS, abdomen	2 (0–5)	1 (1–1)	2 (1–4)	1 (1–1)	2 (0–8)	2 (1–3)	1 (1–3)	1 (1–1)	1 (1–3)	1 (1–1)	0.16^‡^
AIS, spine	0.5 (0–3)	--	2 (2–2)	1 (1–1)	0 (0–0)	--	1 (1–1)	2 (1–2)	2 (2–2)	0 (0–0)	0.08^‡^
AIS, upper extremity	0.5 (0–1)	1 (1–1)	2 (1–2)	1 (1–1)	0 (0–5)	2 (2–2)	1 (1–1)	1 (1–2)	1 (1–2)	0 (0–0)	0.02^‡^
AIS, lower extremity	2 (0–4)	1 (1–1)	2 (2–2)	2 (1–2)	0 (0–4)	2 (2–3)	1 (1–2)	1 (1–3)	1 (1–3)	0 (0–0)	0.19^‡^
AIS, external	1 (0–1)	1 (1–1)	1 (1–1)	1 (1–1)	1 (0–3)	1 (1–1)	1 (1–1)	1 (1–1)	1 (1–2)	1 (1–1)	**0.002**^‡^

*GCS*, Glasgow Coma Scale; *ISS*, injury severity score; *AIS*, abbreviated injury scale

*Two centers were not included in this high-speed subset analysis as they did not capture data on the speed of motor vehicle collision. ^†^*p*-values are from chi-square tests unless noted otherwise. ^‡^Kruskal-Wallis test *p*-value

Median and range unless noted otherwise

### Imaging and outcomes for PTPs involved in high-speed MVCs

There was significant variability in the use of a CT head (12.5–64.3%, *p*<0.001), cervical spine (16.7–75%, *p*<0.01), chest (7.0–83.3%, *p*<0.001), and abdomen/pelvis (0.0–60.0%, *p*<0.001) when comparing trauma centers. There was also variability in the use of MRI (0.0–17.9%, *p*=0.02) (Table 5). Trauma centers C, G, H, I, and K (all Level-I trauma centers) had the highest rates of abdomen/pelvis CT imaging—the scans with the highest radiation dose to the fetus.

**Table 5:** Imaging practices for pregnant trauma patients involved in high-speed (>25 mph) motor vehicle collisions at ten trauma centers

Total, *N*=480	C* (56)	D (8)	E (24)	F (51)	G (129)	H (20)	I (51)	J (71)	K (56)	L (14)	p-value^†^
E-FAST positive	11.5% (6)	0.0%	4.2% (1)	7.8% (4)	0.8% (1)	5.0% (1)	2.2% (1)	1.5% (1)	7.1% (4)	0.0%	**0.05**
CXR	5.7% (3)	0.0%	4.3% (1)	0.0%	0.8% (1)	7.1% (1)	10.0% (4)	2.8% (2)	1.8% (1)	0.0%	0.11
MRI	3.6% (2)	0%	16.7% (4)	15.7% (8)	5.4% (7)	0%	3.9% (2)	7.0% (5)	17.9% (10)	14.3% (2)	**<0.01**
CT, head	35.7% (20)	62.5% (5)	12.5% (3)	15.7% (8)	29.5% (38)	45.0% (9)	31.4% (16)	47.9% (34)	35.7% (20)	64.3% (9)	**<0.001**
CT, cervical spine	39.3% (22)	75.0% (6)	16.7% (4)	27.5% (14)	47.3% (61)	45.0% (9)	35.3% (18)	54.9% (39)	39.3% (22)	64.3% (9)	**<0.01**
CT, chest	30.4% (17)	12.5% (1)	83.3% (2)	5.9% (3)	54.3% (70)	55.0% (11)	25.5% (13)	7.0% (5)	35.7% (20)	35.7% (5)	**<0.001**
CT, abdomen/pelvis	30.4% (17)	0.0%	20.8% (5)	5.9% (3)	59.7% (77)	60.0% (12)	33.3% (17)	11.3% (8)	50.0% (28)	14.3% (2)	**<0.001**

*E-FAST*, extended focused assessment with sonography in trauma; *CXR*, chest radiograph with significant findings; *MRI*, magnetic resonance imaging; *CT*, computed tomography

*Two centers were not included in this high-speed subset analysis as they did not capture data on the speed of motor vehicle collision. ^†^*p*-values are from chi-square tests unless noted otherwise

A similar rate of overall in-hospital procedures was observed, although there was variability in cesarean hysterectomies performed (0.0–75.0%, *p*=0.01). In fact, all the previously mentioned cesarean hysterectomies were performed in this high-speed MVC cohort. Similarly, to the overall MVC population, there were no differences in the rate of fetal delivery (0.0–14.3%, *p*=0.95) and ICU LOS among centers. However, there was variation in overall LOS (1.1–5.7 days, *p*<0.001) and ventilator days (0.0–0.5 days, *p*=0.03), as was seen in the overall MVC population. No differences in mortality were observed among centers (0.0–4.2%, *p*=0.14) (Table 6).

**Table 6:** Outcomes for pregnant trauma patients involved in high-speed (>25 mph) motor vehicle collisions at ten trauma centers

Total, *N*=480	C*	D	E	F	G	H	I	J	K	L	*p*-value^†^
Required in-hospital operations	10.7% (6)	0.0%	4.2%	2.0% (1)	2.3% (3)	15.0% (3)	7.8% (4)	5.6% (4)	8.9% (5)	0.0%	0.15^§^
Tracheostomy	0.0%	0.0%	(1)	0.0%	0.0%	0.0%	0.0%	0.0%	0.0%	0.0%	0.15^§^
Laparotomy	3.6%	0.0%	0.0%	0.0%	0.8%	5.0%	3.9%	0.0%	0.0%	--	0.09^§^
Thoracotomy	0.0%	0.0%	4.2%	0.0%	0.0%	0.0%	0.0%	0.0%	0.0%	0.0%	n/a ^§^
Craniotomy/craniectomy	0.0%	0.0%	0.0%	0.0%	0.0%	5.0%	0.0%	0.0%	0.0%	0.0%	0.09^§^
Vascular/endovascular surgery	0.0%	--	0.0%	0.0%	0.0%	5.0%	2.0%	1.4%	0.0%	--	0.21^§^
Resuscitative hysterotomy/perimortem	1.8%	--	0.0%	0.0%	0.0%	0.0%	0.0%	0.0%	0.0%	--	0.58^§^
Cesarean section											
Cesarean hysterectomy	0.0%	--	0.0%	0.0%	0.0%	0.0%	5.9%	0.0%	0.0%	--	**0.04**^§^
Fetal delivery during hospital stay	5.4% (3)	0.0%	12.5% (3)	5.9% (3)	8.5% (11)	10.0% (2)	9.8% (5)	7.0% (5)	8.9% (5)	14.3% (2)	0.95
Intensive care unit days	0 (0–3)	0 (0–0)	0 (0–8)	0 (0–13)	0 (0–2)	0 (0–6)	0 (0–4)	0 (0–6)	0 (0–4)	0 (0–0)	0.11^‡^
Ventilator days	0 (0–3)	0 (0–0)	0 (0–4)	0 (0–0)	0 (0–0)	0 (0–8)	0 (0–2)	0 (0–1)	0 (0–0)	0 (0–0)	**0.03**^‡^
Length of stay (days)	1 (1–76)	2 (1–2)	1 (1–15)	1 (0–12)	2 (0–65)	1.5 (1–68)	1 (0–5)	1 (0–10)	2 (1–24)	1 (0–6)	**0. 0001**^‡^
Mortality	0.0%	0.0%	4.2% (1)	0.0%	0.0%	0.0%	0.0%	0.0%	0.0%	0.0%	0.14^§^

*Two centers were not included in this high-speed subset analysis as they did not capture data on the speed of motor vehicle collision

^†^*p*-values are from chi-square tests unless noted otherwise. ^‡^Kruskal-Wallis test *p*-value, ^§^Fisher’s exact test *p*-value

## Discussion

Trauma is the leading cause of nonobstetric maternal death, responsible for 20% of maternal deaths in the USA [1, 2, 7, 8, 11]. Expeditious and accurate injury identification is imperative to potentially improve the outcomes of PTPs. However, providers must consider the consequences of radiation exposure to the developing fetus when choosing to perform CT imaging. This multicenter study found more than 50% variability across trauma centers for the rate of CT imaging of the head, cervical spine, chest, and abdomen/pelvis for PTPs. Similarly, in a subgroup of high-speed MVCs, there remained significant variability in the use of CT head, cervical spine, chest, and abdomen/pelvis when comparing trauma centers.

Concern for radiation-induced fetal harm begins around 50 mGy [1]. The amount of radiation exposure is dependent on the CT imaging protocol (e.g., the number of slices in a given area). However, location also matters, a CT scan with a thickness of 10 mm for the head would require >100 scans to equal a cumulative fetal toxic radiation dose, whereas a CT abdomen/pelvis with intravenous contrast followed by delayed imaging or a CT angiography scan followed by venous phase (e.g., multiphase imaging) would each have double the radiation exposure compared to a single phase scan and would surpass the toxic fetal dose of radiation (Table 7) [3, 22–25]. Due to the potential radiation risk to the fetus from CT imaging, PTPs in clinical practice may be evaluated differently than non-pregnant patients. This is evidenced by an American College of Radiology and American College of Obstetricians and Gynecologists survey, where over 73% of respondents noted they alter their CT protocols to account for pregnancy safety. This can be accomplished by shielding the fetus during CT imaging away from the abdomen [12] or adjusting the CT radiation protocols to deliver a lower dosage [6, 19, 26] of ionizing radiation [6, 19, 26]. In fact, the Western Trauma Association recommends the use of radiograph shielding whenever possible [4]. However, none of these national organizations nor the Eastern Association for the Surgery of Trauma or other trauma organizations put forth explicit guidelines regarding when to image PTPs [23]. In addition, few studies exist regarding imaging practices for PTPs [1, 2, 4–14, 16, 18, 27]. Maxwell et al. demonstrated that PTPs underwent CT imaging less frequently compared to non-pregnant patients (48% vs 67%, *p*<0.0001) at their single trauma center [12]. This supports the premise that PTPs are not managed like non-pregnant adults in relation to CT imaging. This current multicenter study further supports this as PTPs involved in MVCs undergo variable CT imaging across trauma centers with rates of CT imaging among centers ranging by 45.7% for CT chest (lowest center 4.48% vs highest center 50.25%) to 63% (lowest center 11.8% vs highest center 75%) for CT cervical spine. Thus, there appears a need for the development of consensus guidelines and best practices to help standardize optimal care for PTPs.

**Table 7:** Fetal radiation exposure in various imaging modalities

Examination type	Estimated fetal dose per examination (mGy)
Chest radiograph (2-view)	0.002
Computed tomography (CT) head	<0.50
CT chest	0.2
CT abdomen/pelvis	29
Multiphase CT imaging (e.g., CT abdomen/pelvis with delayed imaging)	58

The main purpose of CT imaging of trauma patients is to identify life-threatening injuries such as blunt thoracic aortic injuries. This potentially lethal injury may occur even in the absence of plain film radiographic findings [28, 29]. As such, it is recommended to perform a CT with intravenous contrast for all trauma patients with sufficient mechanisms to cause this injury (e.g., high-speed MVC). In contrast, this imaging practice is not recommended for pediatric trauma patients due to the low incidence of blunt thoracic aortic injury. In terms of PTPs, the incidence of blunt thoracic aortic injury has been purported to be quite low as well, with only case reports in the literature [13, 15]. This multicenter study of 729 MVC patients spanning 5 years of data across 12 busy trauma centers found no pregnant patient suffered a blunt thoracic aortic injury or required a thoracotomy. This may call into question the necessity of performing CT chest imaging on all PTPs, at least purely due to the mechanism of injury. Future prospective research is needed to corroborate these findings, but caution should be exercised when deciding to perform a CT chest for PTPs.

## Limitations

This study has many limitations, including those inherent to its retrospective design such as missing data. In addition, pertinent missing variables include baseline adult CT imaging rates for these trauma centers and details regarding the severity of the MVC (i.e., seat belt use, vehicle intrusion, and airbag deployment). In addition, missing data regarding long-term fetal outcomes is an important limitation that merits future prospective research. Also, this study did not account for post-discharge imaging and outcomes. In addition, two of the centers included in the overall study did not record the speed of MVCs, which may have skewed results for the high-speed cohort, although based on the significant variation seen in the overall population of MVCs, this would seem unlikely. Furthermore, trauma patients not specifically evaluated by trauma teams were not included in this analysis. Finally, despite this being a multicenter study, the overall population was relatively small, and all participating trauma centers were located within the same region, thus possibly preventing the generalization of this data. However, one would presume that within a certain region, there might be more uniformity in management practices, which was not demonstrated in this study.

## Conclusion

This multicenter retrospective study demonstrated significant variation in the performance of CT imaging of all body regions (head, cervical spine, chest, and abdomen/pelvis) for PTPs involved in MVCs, the most common mechanism of injury for PTPs. This finding held true even across a cohort of high-speed MVCs. In addition, most outcomes including mortality and in-hospital procedures were similar when comparing trauma centers. Together, these results underscore the importance of developing practice management guidelines for when to obtain specific CT imaging for PTPs, to minimize additive radiation exposure to the developing fetus, while ensuring optimal outcomes.
